# An evolutionary view on the developing prefrontal cortex connectome and its psychopathological networks: Tract tracing and imaging studies linking two distant anthropoid species

**DOI:** 10.21203/rs.3.rs-8755074/v1

**Published:** 2026-02-10

**Authors:** Volker A. Coenen, Alexander Rau, Akiya Watakabe, Henrik Skibbe, Tetsuo Yamamori, Thomas E. Schläpfer, Manuel Czornik, Dora Meyer-Doll, Dominique Endres, Juan Carlos Baldermann, Horst Urbach, Máté D. Döbrössy, Bastian E.A. Sajonz, Marco Reisert

**Affiliations:** University of Freiburg; University of Freiburg; Riken Center For Brain Science; Matsuyama University; CIEM (CentralInstitute for Experimental Medicine and Life Science); University of Freiburg; University of Freiburg; University of Freiburg; University of Freiburg; University of Freiburg; University of Freiburg; University of Freiburg; University of Freiburg; University of Freiburg

**Keywords:** prefrontal cortex, marmoset, human primate, connectomics, psychopathological networks, OCD, Parkinson’s disease, subthalamic nucleus, DBS

## Abstract

The brain has evolved multiple times across evolution; the human prefrontal cortex (PFC) represents its latest developmental addition. Evolution has led to similarities in brain design across species and comparable solutions can be found in different species. The common marmoset shares ancestry with the human primate and serves as a useful model for human PFC build. Previous research approaches on major depressive disorder (MDD) and obsessive-compulsive disorder (OCD) have led to the definition of psychopathological sub-networks including their cortico-subcortical connections. Diffusion tensor magnetic resonance imaging fiber tracking (DTI-FT) is used in humans as a non-invasive substitute for invasive viral tract tracing (AAVaTT) technologies applied in non-human primates. We here compare DTI-FT (N = 1000) and AAVaTT (n = 52) in humans and marmosets, respectively, to reveal interspecies anatomical and functional distinctions of PFC connectivity models and putative effects on dysfunctional networks relevant for MDD and OCD.

## Introduction

**Figure 1:**
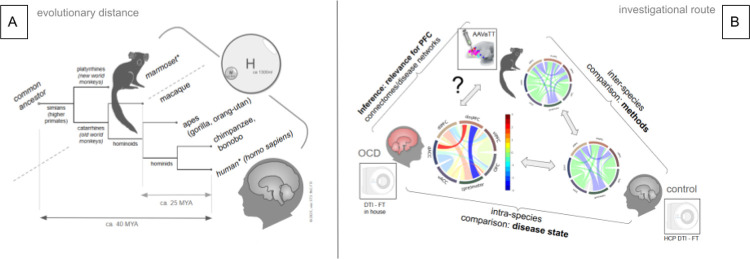
Development of anthropoid species and the investigational route taken. **A, evolutionary distance**. The dotted line separates the new world and old world monkeys. The arrow indicates evolution from a joint ancestor to the modern human primate brain. The marmoset as such represents a distant evolutionary relative of the human primate (inspired by (Homman-Ludiye and Bourne 2021)). Spheres indicate brain volume proportions. **B, principal investigational strategy**. Inter-species comparison allows for a comparison of methods (ATTS vs. DTI). Intra-species (human) comparison with a disease state for assessment of plausibility. Results can be extrapolated towards an appreciation of interspecies validity (anatomy and function) of PFC connectomes and psychopathological networks. Legend: MYA, million years apart; *compared species, marmoset (M), human (H); PFC; prefrontal cortex; OCD (obsessive-compulsive disorder).

The brain is the most complex organ and its evolution is the result of numerous steps of refinement. The latest evolutionary addition to the brain is the prefrontal cortex (PFC). Assuming that evolutionary pressure of the brain’s design shares certain anatomical similarities, one can expect that similar solutions subserving similar functions can be found in different species during evolution. Brain development continues in side-branches of the evolutionary tree, one of which lead to humans. This creates similarities that allow inferences on joined ancestors and therefore on joint anatomy. It is therefore conceivable to look for anatomical similarities in nonhuman primates (NHP) which have a common ancestor with humans (Fig. 1A).

The common marmoset (Callithrix jacchus) is a new world primate species that has been used to model development of the human brain and many aspects of its function. Supposed similarities in PFC organisational patterns, the short reproduction cycle, and its social behavior render the marmoset a valuable neuroscientific model for comparative studies in neuropsychiatric disorders. A recent addition to the view on psychopathologies in humans is the definition of disease-related (sub-) networks which allows for disentangling phenotypical and taxonomical aspects in the explanation of distinct disease features. For obsessive-compulsive disorder (OCD) (and major depressive disorder (MDD) (Coenen et al. 2020)), advanced neuroimaging has facilitated the definition of such subnetworks (Li et al. 2018; Shephard et al. 2021). Following the tenets of affective neuroscience (Panksepp 1998) and extending its viewpoints, we argue that emotions primarily arise subcortically and are only secondarily modulated and regulated on the cortical level. Thus, subcortically located systems initially realize distinct emotional responses, making a cortico-subcortical interface as integral part of such networks indispensable (Coenen et al. 2020, 2022). Moreover, target regions of primarily descending pathways (projection pathways) are realized as deep-seated network hubs (Coenen et al. 2020), as such allowing for a therapeutic access to larger parts of psychopathological networks in humans via Deep Brain Stimulation (DBS).

Tractographic approaches are required to reveal connections of cerebral brain regions in humans and diffusion tensor magnetic resonance imaging-based fiber tractography (DTI-FT) is employed in vivo since long-range tract tracing (TT) is invasive and therefore not feasible. DTI-FT can describe underlying altered connectivity also in disease models, but has important limitations with respect to directionality, low resolution, and lack of transmitter specificity and for the detection of synaptic connections between neuronal structures. We have previously (Coenen et al. 2020) defined cortical and subcortical sub-network extensions for OCD and MDD using the DTI-FT technology in a normative sample of the human connectome project (HCP) employing a corticopetal ordering heuristic. However, because of the indirect nature of such a connectomic network model, it appears justified to search for the constituents of these networks in other less developed non human primate species, thereby relying on classical tract tracing experiments to understand how well human DTI-FT depicts the network anatomy (Coenen et al. 2023) but moreover what the evolutionary distinctions consist of. Most previous analyses were performed comparing macaque monkey and human anatomy (Croxson et al. 2005; Milham et al. 2018) which are only 25 Mio years apart in their evolution (Consortium et al. 2007) (Fig. 1A). These studies were geared towards observing macaque and human similarities but also evolutionary distinctions. We have here used prefrontal viral (adeno-associated virus = AAV) injections in the common marmoset (n = 52, left, prefrontal locations) to analyse its complex PFC connectome and to compare these results with a rendition of the human PFC connectome (HCP, n = 1000), based on DTI-FT. On the one hand, marmoset’s PFC hard-wiring - as detailed with AAVaTT - might serve as the blueprint for more qualitative human DTI-FT and anatomically derived descriptions of the PFC connectome. On the other hand, distinct inter-species quantitative detailing might explain functional differences and even more so with respect to psychopathologies specific to humans (Fig. 1B). To our knowledge, there has so far been no attempt to directly and holistically compare the PFC connectivity of human and marmoset in species-specific common spaces, at the same time detailing fiber anatomical routes, cortico-cortical and cortico-subcortical connections and their quantitative contributions to our current sub-network descriptions in psychiatric diseases.

## Methods

**Figure 2:**
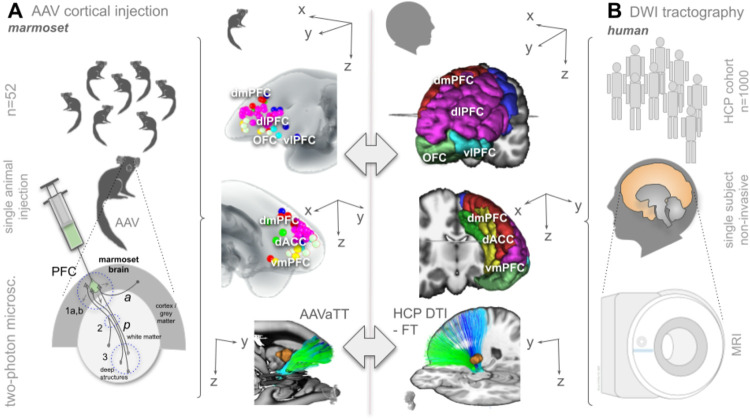
Principal workflow. Panel A, tract tracing injection study in n=52 female common marmosets. Injection regions are rather homogeneously dispersed over the left prefrontal cortices. Anterograde tomographic tract tracing streamline tractography (ATTS) is used to create fiber streamline reconstructions (Coenen et al. 2023). Panel B, sub-cohort from the human connectome project (HCP) data repository (n=1000) to create group level diffusion tensor magnetic resonance imaging (MRI DTI) renditions of streamlines. (*Legend: AAV, adeno-associated virus; PFC, prefrontal cortex; dmPFC, dorsomedial PFC; dlPFC, dorsolateral PFC; vlPFC, ventrolateral PFC; vmPFC, ventromedial PFC; OFC, orbitofrontal cortex.)*

Our principle approach is shown in Fig. 2.

### Ethics

#### Marmoset:

All experiments followed the NIH “Guide for the Care and Use of Laboratory Animals” (NIH Publication No. 80 − 23, revised 1996) and the Japanese Physiological Society’s “Guiding Principles for the Care and Use of Animals in the Field of Physiological Science.” Procedures were approved by the Experimental Animal Committee of RIKEN (protocols H26-2308 ~ W2022-2-010). The authors state that all efforts were made to minimize animal suffering and to reduce the number of animals used.

#### Patients:

Patients with OCD who previously had received bilateral slMFB-DBS (Meyer et al. 2022; Coenen et al. 2025) were selected for analysis, if they gave informed consent to our DBS registry that adheres to the principles of the Helsinki Declaration and received approval from institutional review board (Ethics committee of the University of Freiburg; no.21–1274).

### Data

#### Marmoset

**Figure 3:**
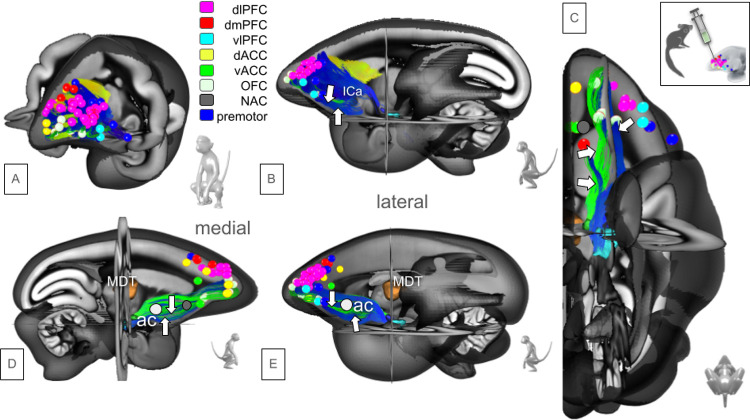
Detailed injection sites (marmoset) and their resulting projection fibers. A, overview of all n=52 injections in left PFC; Color-coding of fiber tracts: see Figure 3–5. B, lateral view showing all fibers; C, view from inferior, detailing injections which contribute to sub-ICa branch (white arrows); D-E; sub-ICA branch (white arrows) shown from medial (D) and lateral (E). White sphere indicates anterior commissure (ac). Legend: ACC, anterior cingulate cortex; dACC, dorsal ACC; vACC, ventral ACC; ICa, internal capsule, anterior limb; OFC, orbitofrontal cortex; NAC, nucleus accumbens; PFC, prefrontal cortex; dlPFC, dorsolateral PFC; dmPFC, dorsomedial PFC; vlPFC, ventrolateral PFC; MDT, mediodorsal thalamus.

Our dataset includes 52 *Callithrix jacchus* (common marmosets), each receiving a unique prefrontal injection of an AAV-based fluorescent anterograde tracer. The standard mix contained AAV1-Thy1S-tTA, AAV1-TRE-clover (GFP derivative), and AAV1-TRE3-Vamp2-mTFP1 (cyan fluorescent protein). Later experiments also included AAV2retro EF1-Cre. Plasmids for these vectors were deposited in Addgene. Injections were performed under general anesthesia using motor-driven pressure injections with a glass micropipette. Injection distribution is shown in Fig. 3.

Animals were kept alive for four weeks before brain harvesting and scanning via serial two-photon tomography (STPT), producing distortion-free 50μm slices (1.35μm in-plane resolution). Additional Nissl staining and brightfield images were acquired post-STPT. Injection sites covered the left prefrontal cortex, including the medial prefrontal cortex (mPFC) and orbitofrontal cortex (OFC). Imaging data was normalized to a common reference space. A convolutional neural network (CNN) segmented axons in full-resolution images, and axonal density maps (100μm isotropic resolution) were computed. For details, see Skibbe et al. (29) and Brain/MINDS (30, 73).

#### Human Connectome Project

This study utilizes data from the Human Connectome Project (HCP 3T) (HCP database). We analyzed dMRI, data from 983 participants (mean age 29 ± 7 years) from the HCP (Q1:S1–4) dataset. The imaging resolutions were 1.25 mm isotropic for diffusion-weighted MRI. Each gradient table contained ~ 90 diffusion-weighting directions and interspersed b = 0 acquisitions per run. Diffusion weighting was applied across three shells (b = 1000, 2000, and 3000 s/mm^2^) with an equal number of acquisitions per shell (18 b0, 3 shells with 90 directions each). Preprocessing followed the HCP pipeline (Glasser et al., 2013), including spatial normalization to MNI standard space. Results were displayed on an average T1w image and surface created in VBM8 from 550 healthy IXI-database participants (IXI dataset), aligned to MNI ICBM152 2009b asymmetric space.

#### Inhouse Datasets

Demographic information about patients can be found in supplements **(sTable 1)**.

MRI was performed with a 3 Tesla scanner (MAGNETOM Prisma, Siemens Healthcare, Erlangen, Germany) with a 64-channel head and neck coil. T1-weighted (T1w) images were acquired with a three-dimensional (3D) magnetization-prepared 180° radio-frequency pulses and rapid gradient-echo (MP-RAGE) sequence (repetition time: 2500 ms, echo time: 2.82 ms, flip angle: 7°, TI = 1100 ms, GRAPPA factor = 2, 1.0 mm^3^ isotropic voxels, 192 contiguous sagittal slices). The DTI/DMI sequence was acquired with the following parameters: axial orientation, 42 slices, voxel size 1.5 × 1.5 × 3 mm^3^, TR 2800 ms, TE 88 ms, bandwidth 1778 Hz, flip angle 90°, simultaneous multi-band acceleration factor 2, GRAPPA factor 2, 65 diffusion-encoding gradient directions, 15 non-diffusion weighted images, 2 × 58 images with b-factors 1000 and 2000 s/mm^2^; acquisition time was 6:22 min.

Assignment of cortical and subcortical regions to specific psychopathological sub-networks

The composition of psychopathological sub-networks is shown in Table 1.

### Analysis

#### Anterior tract tracing streamline (ATTS) tractography in the marmoset

Tracer load maps lack inherent orientational information, making visualization of projection pathways challenging. To address this, we employ a variational method that enhances these maps with directional information, enabling the use of standard streamline visualization techniques. We solve the following partial differential equation

$$\nabla \cdot \left(\sigma \left(r\right)\nabla \varphi \left(r\right)\right)+\alpha \varphi \left(r\right)-\delta \left(r-r_{0}\right)=0$$

which is close to the Poisson equation with an additional term *α φ* (*r*). The position *r*_0_ can be interpreted as the location of the tracer injection. The term *σ* (*r*) is associated and proportional to the tracer load map and may be interpreted as an axonal conductivity term. Solving the equation for *φ* (*r*) and computing the flux *j*(*r*) = *σ* (*r*)∇ *φ* (*r*) yields a plausible proxy for the mean underlying axonal direction. We assume von Neumann boundary conditions, i.e. no flux across the boundaries. The resulting flux can be interpreted in two ways: as the electric current of an electrostatic problem, where the injection site serves as the current source and the tracer load is the electric conductivity. The additional term *α φ* (*r*) is responsible for a small leakage current present in the whole volume. In the second interpretation, the flux may be interpreted as the mean of the local tangent distribution of a random Brownian walker, where the path costs are dependent on the tracer load via the Lagrangian *L*(*s*) = *∮ dt σ* (*s*(*t*)) (*ds/dt*), where *s* is a random path. The above Poisson equation is just the steady state solution of the corresponding Fokker-Planck equation. We discretized the equation by an ordinary finite-element scheme (on a matrix with 100μm isotropic resolution) and solved it with the minres algorithm as implemented in MATLAB. Based on the flux, we can now perform ordinary streamline integration techniques (like used in diffusion-weighted MRI-based tractography) to obtain visual appealing representations of the axonal pathways.

#### Diffusion-based global tractography in the human

Tractography was carried out using a global approach (Reisert et al., 2011). In contrast to local deterministic or probabilistic methods, global tractography does not depend on predefined seed or target regions, thereby avoiding biases from predetermined starting and stopping points. Instead, anatomical specificity is introduced only at a later stage of the analysis. In a first step, whole-brain connectomes were reconstructed from each participant’s diffusion-weighted MRI data. Regions of interest (ROIs), defined in atlas space either by MNI coordinates or explicit masks, were then warped into individual subject space and used to extract specific bundles from the reconstructed connectomes. As the tracking itself is performed independently of the ROIs, the procedure is free from biases imposed by prior tract definitions. Global Gibbs tractography (Reisert et al., 2011) generates streamlines using a random point process and optimizes their configuration to best explain the diffusion MRI signal. This approach yields fiber configurations that provide a maximum-likelihood explanation of the measured data (Fillard et al., 2011). It has been shown to be robust to noise, and fiber densities are directly related to the underlying signal (Fillard et al., 2011; Reisert et al., 2011; Schumacher et al., 2018). We followed the publicly available implementation described by Reisert et al. (2011), using the “dense” preset of the toolbox, which automatically adapts tracking parameters to data resolution and diffusion signal properties. For HCP datasets, this typically produces ~ 150,000 streamlines. To further enhance reproducibility, we applied an accumulation strategy as proposed by Schumacher et al. (2018). After the initial cooling-down phase, the temperature was reset to 0.1, followed by an additional 10^7^ iterations. This procedure was repeated across five rounds, and the resulting tractograms were combined into a final reconstruction that was approximately five times larger than the initial one. For a single HCP participant, this resulted in tractograms containing around 800,000 streamlines. The reconstruction was restricted to white-matter regions defined by the parcellation accompanying the HCP dataset, with the resulting white-matter mask slightly dilated to include the white–gray matter transition zone.

#### Parcellation of the prefrontal cortex

Due to its specialization in distinct cognitive and affective functions and its evolutionary preservation, we parcellated the prefrontal cortex into the OFC, dorsolateral PFC (dlPFC), ventrolateral PFC (vlPFC), dorsomedial PFC (dmPFC), dorsal anterior cingulate cortex (dACC), ventral anterior cingulate cortex (vACC), and (pre)motor cortex. For humans, we used the extended HCP multimodal parcellation atlas (Huang et al. 2022) to build such a parcellation (according to Table M1). We followed a similar approach for the marmoset and assigned each injection site to one of the seven parcels. To this end, injection locations from the Brain/MINDS database were manually mapped to the corresponding prefrontal subdivisions according to the nomenclature provided in the Brain/MINDS 3D digital marmoset brain atlas (Skibbe et al. 2023). The Brain/MINDS atlas adopts the Paxinos et al. stereotaxic atlas of the marmoset brain (Paxinos et al., 2012) as its reference framework, and we used this Paxinos-based nomenclature for consistent parcel assignment across injection sites.

#### Definition of the subcortical regions

For humans, we defined the set of subcortical parcels based on Ilinsky’s Human Motor Thalamus atlas (Ilinsky et al. 2018) and additional MNI-based coordinates. Specifically, we included the subthalamic nucleus (STN), substantia nigra (SN), red nucleus (Ruber), mediodorsal thalamus (MD), ventrolateral thalamus (VL, combining dorsal and ventral divisions), and the ventral tegmental area (VTA). The VTA parcel was defined by an MNI coordinate sphere centered at (−6, − 12, − 8) with a 2mm radius. These regions were used as waypoints to compute subcortical structural connectivity profiles.

For the marmoset data, the subcortical parcellation was performed using the Brain/MINDS 3D digital marmoset brain atlas (Skibbe et al. 2023), which provides high-resolution delineations of subcortical structures. We extracted the homologous parcels corresponding to the human analysis, including STN, SN, red nucleus, MD, and VL thalamus. The VTA was defined according to the approach established in our previous marmoset study (Coenen et al. 2023), ensuring consistency of the ROIdefinition across species.

#### dMRI based Structural Connectivity

Based on whole-brain tractography (as described above), we computed streamline counts between cortical and subcortical parcels. Cortical regions were defined using the fused PFC parcellation, while subcortical regions comprised STN, SN, red nucleus, MD, VL, and VTA as described above. A streamline was assigned to connect two parcels if it traversed both regions. This procedure enabled the systematic quantification of structural connectivity both within the cortex (cortex–cortex) and between cortical and subcortical regions (cortex–subcortex). To warp the parcellation from group space to individual space, we used the shipped warp fields for the HCP cohort. For our in-house datasets, we followed the robust approach proposed in (Reisert et al. 2022) using deep learning and the ANTs toolbox (Avants et al. 2011). Finally, the streamline counts were normalized by the Sinkhorn–Knopp algorithm (Sinkhorn 1964), balancing its row and column sums to produce a normalized connectivity profile suitable for direct comparison across injection groups and target areas.

#### Tracer based Structural Connectivity

We start by normalizing each individual tracer image using its Euclidean (L_2_) norm, thereby removing absolute intensity differences between injections. Next, tracer images were grouped according to the seven PFC parcels from above (see Table M1), and within each cortical injection group, the normalized volumes were summed voxel-wise to produce a single composite “sum image” representing that group’s overall projection pattern. From each sum image, the mean tracer signal was then measured within every predefined subcortical region to yield raw connectivity strengths linking each cortical injection group to each subcortical target. Finally, the resulting connectivity matrix was converted into a doubly-stochastic form via the Sinkhorn–Knopp algorithm.

## Statistical Analysis

For the human dataset, we compared in-house acquired data from healthy controls (HC) and an obsessive–compulsive disorder (OCD) cohort. With a small albeit severely affected cohort of n = 12 our analysis might be regarded as hypothesis generating or explorative. The values subjected to statistical testing were *dMRI*-based structural connectivity estimates (cortex–cortex and cortex–subcortex) obtained after Sinkhorn normalization. Group differences were assessed using two-sample *t*-tests without inclusion of covariates. To control for multiple comparisons, Bonferroni correction was applied, and results were considered significant at *p* < 0.05. The analyses were implemented in MATLAB R2021 using the **fitlm** function. For visualization, the resulting *t*-values were displayed in a chord chart, with color coding used to indicate effect direction and magnitude.

## Results

**Figure 4:**
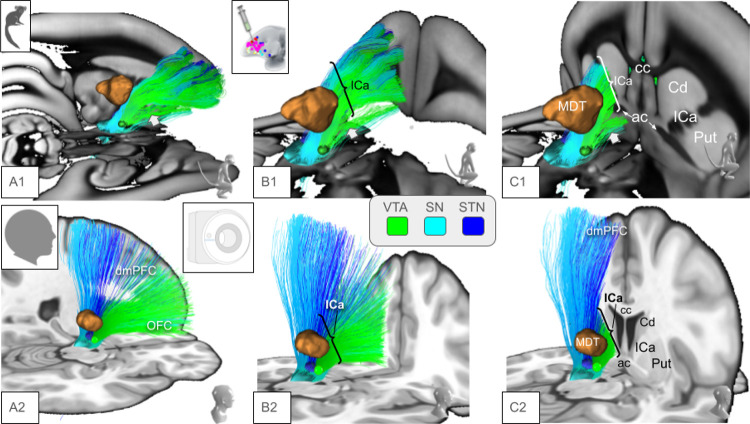
Detailed interspecies comparison of prefrontal fiber anatomy in 3D. Color coding indicates individual targets of subcortical fiber routes (inset). **Upper panel (A-C) marmoset:** White arrow indicates sub-ICa branch of PFC projection fibers. **Lower panel (D-F) human:** entire PFC fibers follow ICa on their subcortically aiming route. *Legend: ICa, anterior limb of internal capsule; cc, corpus callosum; ac, anterior commissure; Cd, caudate nucleus; MDT, mediodorsal thalamus; Put, putamen; OFC, orbitofrontal cortex; dmPFC, dorsomedial prefrontal cortex*.

**Figure 5:**
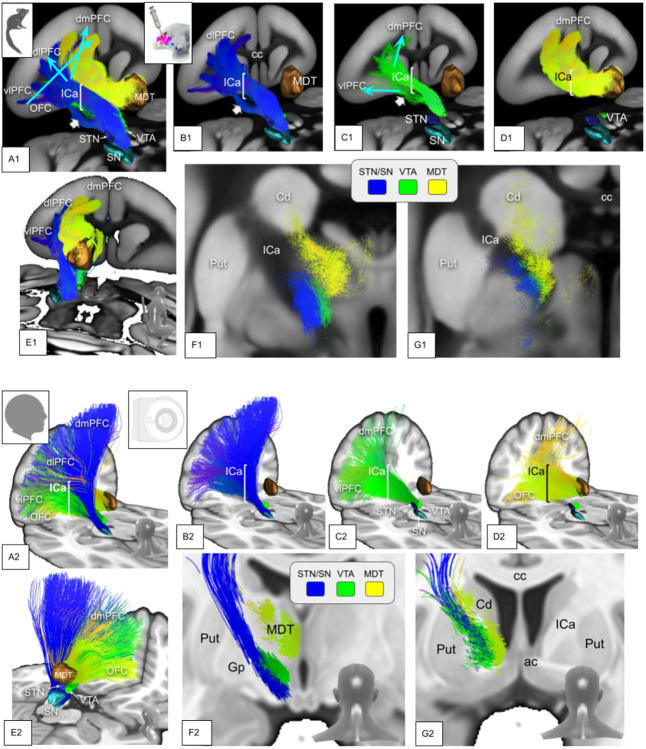
Interspecies comparison of topographic PFC fiber anatomy (3D). Upper panel (A1–G1), marmoset: A1–C1, White arrows indicate a branch of PFC fibers disjunct from ICa (sub-ICA branch). This branch consists of fibers reaching STN/SN and VTA (B1–D1). Turquoise arrows indicate the shift of fiber trajectories towards human PFC anatomy (lower panel). **Lower panel (A2–G2), human:** PFC - subcortical projections follow the ICa. Fibers (green) which travel from vlPFC/OFC undercross MDT-targeted fibers from lateral to medial on their way to the VTA. *Legend: Cd, caudate nucleus; ICa, anterior limb of the internal capsule; Put, putamen; Gp, globus pallidus; cc, corpus callosum; MDT, mediodorsal thalamus; ac, anterior commissure; fx, fornix; STN, subthalamic nucleus; SN, substantia nigra; VTA, ventral tegmental area*.

**Figure 6:**
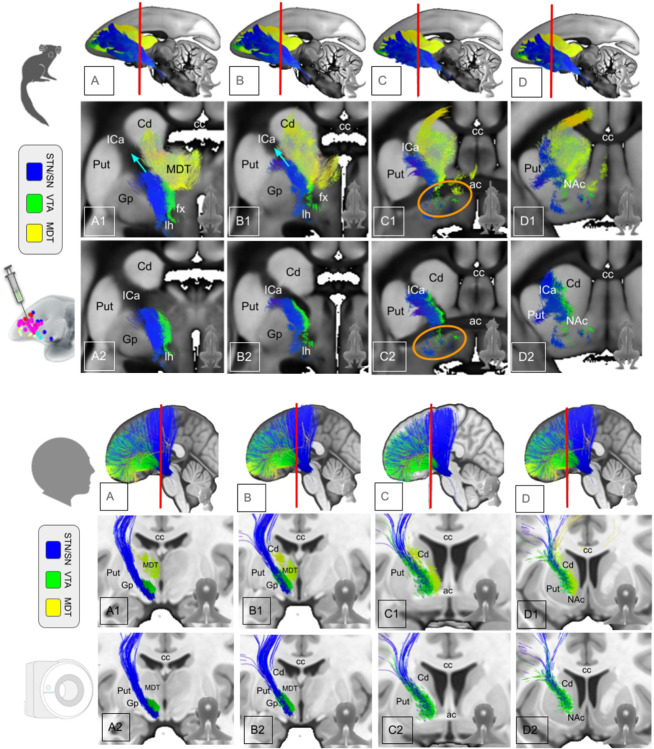
Interspecies comparison of the anterior limb of the internal capsule (ICa). A-D indicates the caudal to anterior progression of coronal slides in A1–D2. A1–D1, include thalamus (MDT) receiving projections (yellow). A2–D2, same as above but without MDT projections. Topographically, marmoset and human share similar patterns of fiber organization. One distinction is a somewhat separated sub-ICa fiber branch in the marmoset, consisting of all projections (STN/SN, MDT, VTA) and originating in basal PFC projections. Such a sub-ICa branch is not observed in humans. **Upper panel, marmoset:** A1 & B1, turquoise arrows indicate direction of development of STN-bound projections during evolution. Orange circles in C1 and C2 exemplarily indicate the sub-ICa branch of PFC fibers effectively under AC. **Lower panel, human:** the entire PFC - subcortical projections follow ICa. *Legend: Cd, caudate nucleus; NAc, nucleus accumbens; ICa, anterior limb of the internal capsule; Put, putamen; Gp, globus pallidus; lh, lateral hypothalamus; cc, corpus callosum; MDT, mediodorsal thalamus; ac, anterior commissure; fx, fornix; STN, subthalamic nucleus; SN, substantia nigra; VTA, ventral tegmental area*.

**Figure 7:**
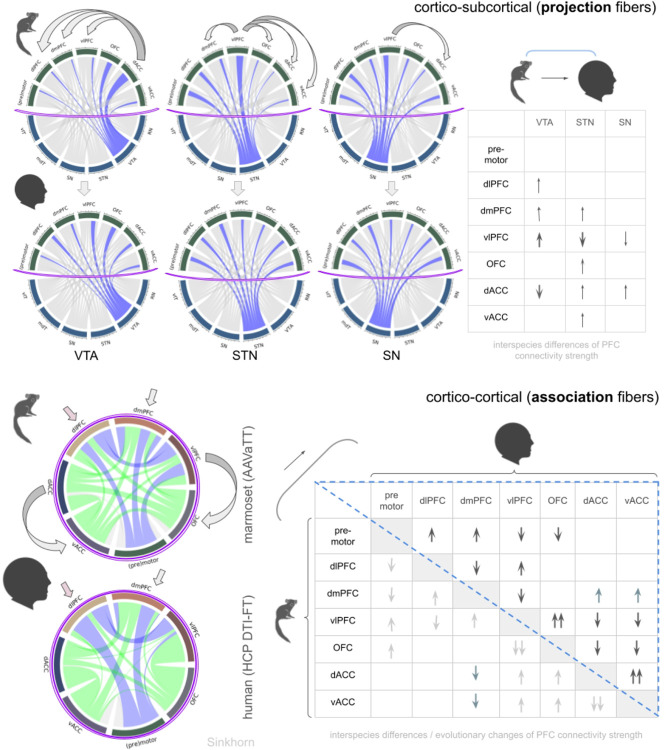
Differential quantitative evaluation of marmoset and human PFC connectomes. Cord plots show individual strength of connections (widths). Normalisation of data according to the Sinkhorn algorithm (Marshall and Olkin 1968). **Upper panel, cortico-subcortical connections** (projection pathways): Purple equators indicate segregation between cortical and subcortical regions. Visually, the connectivity pattern to subcortical structures looks similar between species, though a closer inspection reveals differences (curved arrows indicate interspecies transients). The table indicates relevant connection strength changes from marmoset to human. Most differences appear for VTA and STN (details in result section). **Lower panel, cortico-cortical PFC connections** (association pathways). Again, a very similar interspecies connection pattern. Table evaluates significant differences (direction: marmoset -> human, upper right triangle, blue). Increased connectivity is detected between OFC/vlPFC and dACC/vACC (details in results section). *Legend: vlT, ventrolateral thalamus; mdT, mediodorsal thalamus; VTA, ventral tegmental area; STN, subthalamic nucleus; SN, substantia nigra; RN, red nucleus; PFC, prefrontal cortex; dlPFC, dorsolateral PFC; dmPFC, dorsomedial PFC; vlPFC, ventrolateral PFC; OFC, orbitofrontal cortex; dACC, dorsal anterior cingulate cortex; vACC, ventral anterior cingulate cortex*.

**Figure 8:**
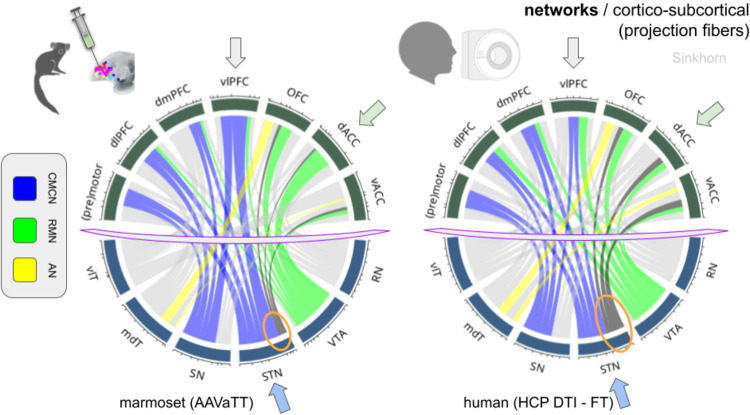
Three sub-networks (control/reward/affect) related to major depression and obsessive-compulsive disorder in a side-by-side comparison highlighting the differing interspecies contributions in fiber connection strength. Arrows indicate regions where most inter-species differences emerge. Sub-networks can be detected in both species. Projection pathways shown, only. Cognitive motor control network (CMCN, control, blue); reward maintenance network (RMN, reward, green); affect network (AN, affect, yellow). Orange circles indicate the changing role of subthalamic nucleus-bound projections between marmoset and human, respectively. For details see text. Note the qualitatively very similar appearing networks which, however, differ on the quantitative level. Cord plots with individual strength of connection (width). Normalisation of data according to the Sinkhorn algorithm. *Legend: vlT, ventrolateral thalamus; mdT, mediodorsal thalamus; VTA, ventral tegmental area; STN, subthalamic nucleus; SN, substantia nigra; RN, red nucleus; PFC, prefrontal cortex; dlPFC, dorsolateral PFC; dmPFC, dorsomedial PFC; vlPFC, ventrolateral PFC; OFC, orbitofrontal cortex; dACC, dorsal anterior cingulate cortex; vACC, ventral anterior cingulate cortex*.

**Figure 9:**
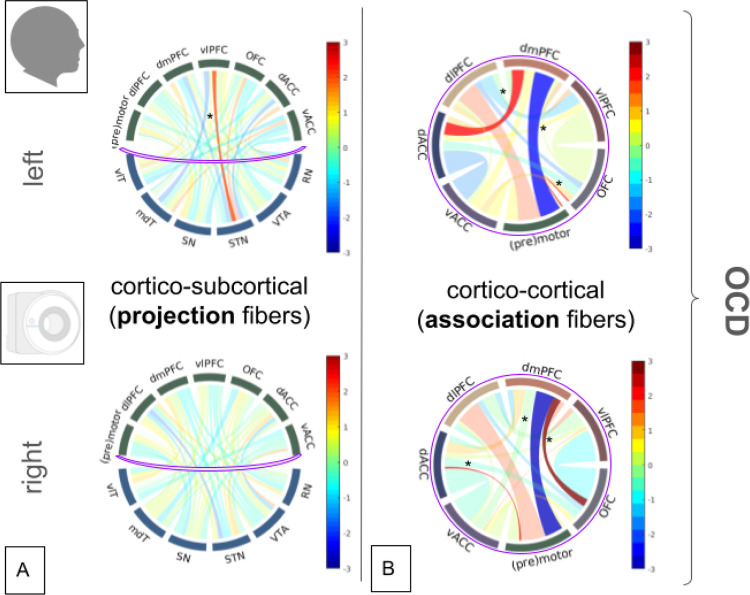
Cord plots of disease-specific differential connectomes for treatment-resistant (OCD) from a patient cohort treated with DBS as a comparison with normal control PFC connectome. A, cortico-subcortical connections (projection fibers). B, Cortico-cortical connections (association fibers). Cord plots show individual connection strength (width). Human, DTI-FT. Normalisation of data according to the Sinkhorn principle. Differences for projection fibers (A, cortico-subcortical) in OCD dominantly concern association projections (B, intra-cortical). Asteriks (*) indicates significance (p<0.05) before a correction for multiple comparisons). *Legend: mdT, mediodorsal thalamus; vlT, ventrolateral thalamus; VTA, ventral tegmental area; STN, subthalamic nucleus; SN, substantia nigra; PFC, prefrontal cortex; dlPFC, dorsolateral PFC; dmPFC, dorsomedial PFC; vlPFC, ventrolateral PFC; OFC, orbitofrontal cortex; dACC, dorsal anterior cingulate cortex; vACC, ventral anterior cingulate cortex*.

The results of this work are essentially summarized in Figs. 3–9.

### Topographic fiber anatomy:

#### Quantitative PFC connectivity to subcortical structures (projection) and other PFC regions (association)

*Interspecies* comparison of projection and association fibers qualitatively show similar patterns. For STN and SN, the vlPFC appears to play a dominant role in marmoset which is given up in humans towards a more homogeneous PFC connectivity. For VTA-bound connections, the predominant role of the OFC (marmoset) is given up for a more homogeneous connection to the entire prefrontal cortex, especially vlPFC, dmPFC and dlPFC (humans). For details, see Fig. 7.

#### Differential PFC connectivity contribution to (disease) sub-networks (dup: abstract ?)

The network directed analysis reveals a principal *contribution of projections to most sub-networks relevant for MDD and OCD*. Such connectivity is already present in the marmoset and qualitative patterns look similar between species (Fig. 8). Connection strength changes between species are driven by altered connectivity between vlPFC, dACC and STN. The vlPFC shows less strong connections to STN and SN in humans. In turn, vACC, dACC, dmPFC and OFC show stronger connectivity to STN in humans. The ventral tegmentum is less connected to dACC in humans than in marmoset but shows a stronger connection to the vlPFC. In sum, PFC regions which in the marmoset are more closely connected to the VTA as subcortical basis of the *reward maintenance sub-network (RMN)* become more closely associated with the STN as the dominating structure of the *cortical/motor control sub-network (CMCN)* in humans. Connectivity of the *affect sub-network (AN)* remains grossly unaltered. The default mode sub-network (DMN) extends outside the PFC and was thus not regarded here.

#### Disease-specific alterations of connectivity

Numerical results can be found in supplements (sTable 2). The differential analyses of alterations of connectivities (in humans) are specific for OCD (Fig. 9): Cortico-subcortical pathways show increased connection strength between vlPFC and STN on the left. No effects are detected for OCD on the right. *Cortico-cortical connections (association fibers)*: Increased connection strength is seen between dmPFC and dACC on the left and dmPFC with OFC on the right. OFC shows increased connectivity with the premotor region on the left. Decreased connectivity of dmPFC is found with the premotor region, bilaterally.

## Discussion

### Topographic fiber anatomy

We have here used two distinct technologies - AAVaTT injections (Fig. 3, n = 52, marmoset) and HCP DTI fiber tractography (n = 1000, human) - to compare PFCs connectomic anatomy in species-specific common spaces. A comprehensive side-by-side description of the human and the marmoset PFC connectomes including their detailed anatomy has not been published previously. By looking at these two evolutionary distantly related anthropomorphic species - ca. 40 MYA - insights into the development of the PFC as (potentially) the most important brain region of the human cortex (and the region that mostly distinguishes us from other primates) are gained. Evolutionary routes of development are interpreted against the background of distinct functioning of marmoset and human PFC. Developing more complex connectomic anatomy is reflective of developing more complex PFC functions. Despite the use of distinct techniques to investigate the PFC connectomes, interspecies comparison looks quite similar but only after the application of the Sinkhorn normalisation (sFigure1, supplements) (Marshall and Olkin 1968). In reversing the argument, we take the alikeness of the result as an indicator that comparing two species while applying distinct analysis techniques (AAVaTT vs. HCP DTI - FT) is justified. We try to find further evidence by investigating the human connectome for disease-specific alterations (Fig. 1B).

The marmoset is not a precursor of the human primate but both species have a common ancestor (Fig. 1A) and there is a chance that effects of evolution can be inferred by comparing interspecies PFC anatomy and connectomes. While the principles of detailed organization of PFC projections - as viewed in ICa anatomy - connectivity appear to be conserved in anthropoid old world monkey species (Jbabdi et al. 2013), we have found here some differences in tract organization (Fig. 4). The composition of the ICa is in principle similar in marmosets as most fibers from more rostral and dorsal portions of the PFC use this structure as pass-through to deeper residing structures (thalamus, basal ganglia, brainstem). However, a sub-ICa branch of projection fibers traverses the PFC more basally and under the anterior commissure. Such a branch is not found in humans. However, we can not be entirely sure if such a branch in humans does simply not exist or if it just escapes the DWI detection threshold (false negative) (Jbabdi et al. 2013)Jbabdi and co-workers have described a small amount of basal vmPFC fibers taking a similar route in the macaque (old world, Fig. 1A) monkey. They have interpreted them as part of IC fibers which are routed through parts of globus pallidus and putamen (Jbabdi et al. 2013). The significance of such aberrant fibers is not clear but might simply represent a developmental step with a further integration of basal PFC fibers into the ICa on a trajectory of development towards a more complex human PFC. It is not entirely clear what the evolutionary advantage of channeling all PFC fibers through ICa might be. However, a clearly defined ICa has been linked to the evolutionary development of a more advanced PFC (Coizet et al. 2017) and therefore with an integration of working memory, cognition and emotion as such allowing complex flexible behavior in anthropoid species. Besides these potentially separated basal forebrain projections, the principles of ICa organization appear to be present as early as in the marmoset: STN/SNR bound projections reside in the lateral aspect of ICa while bidirectional fibers subserving OFC and MDT reside in the medial ICa aspect. The most inferior projections appear to be VTA-bound projection fibers (Fig. 5,6). Similar organization has previously been described (Jbabdi et al. 2013; Nanda et al. 2017; Safadi et al. 2018; Avecillas-Chasin et al. 2021) and additionally with applying a similar cortico-petal heuristic (like we do here) in humans and marmoset (Coenen et al. 2020, 2023). We will discuss below that the relative amount of fiber projections from specific brain regions (e.g. vlPFC) to STN and VTA have changed during species specific development. This potential evolutionary shift of projection fiber balance (Fig. 5, upper panel, A1&B1) might explain the gradual change of the trajectory of STN bound projections residing in human ICa in more lateral and superior positions (Fig. 5, lower panel, A1&B1, turquoise arrows), thereby subserving the development of fiber projections toward dlPFC and dmPFC. The three-dimensional evaluation indicates a similar pattern but now on the cortical level (Fig. 6, upper panel, A1 & B1, turquoise arrows).

#### Quantitative PFC connectivity to subcortical structures (projection) and other PFC regions (association)

The development and re-routing of STN connectivity indicates a more pronounced role of the STN in humans as an advanced but integral part of the PFC. This functional position as an important hub in the *cortical/motor control network* (CMCN) might also explain why DBS of the STN in Parkinson’s disease has such dramatic therapeutic effects on the one hand (Deuschl et al. 2006; Schuepbach et al. 2013), but on the other hand - and under certain circumstances - can elicit symptoms of frontal lobe dysexecutive syndromes (Mosley et al. 2018, 2019; Bucur and Papagno 2022). The trajectory of the interspecies shift of STN connectivity away from vlPFC to more medially located regions (vmPFC, dACC, vACC) aims spatially at a location coinciding with a recently described hub region (MPFC) of the human brain’s meta loop. This hub is linked to higher cognitive functions, including mind wandering and theory of mind (ToM) (Weiller et al. 2025) and as such with advanced remits of the PFC, typical for the human primate. Conversely, VTA-bound connectivity appears to shift towards vlPFC but also towards dmPFC and dlPFC. A clear reduction of connectivity of dACC to VTA occurs. This is remarkable, since the dACC has been given a role in foraging-like decisions (Shenhav et al. 2016a, b; Shenhav and Karmarkar 2019), a role that subcortically is executed by the reward system and thus with the VTA as an integral part (Wise and Bozarth 1984; Schultz et al. 1997; Kalivas and Nakamura 1999; Coenen et al. 2020). In such context, an increased dACC-STN connectivity once more highlights the STN’s importance in the delayed decision making process under (human) PFC control (Frank et al. 2007; Ballanger et al. 2009; Zeng et al. 2021; Schüller et al. 2023). The overall similarities of intracortical PFC connections (association) between marmoset and human are also striking (Fig. 7), especially since our interspecies investigations utilize entirely different techniques. However, quantitative interpretation of cortico-cortical projections indicate a developmentally inferior role of vlPFC in favour of dlPFC and dmPFC in humans (Fig. 7, lower panel). As such, the cortico-cortical results do point in a similar direction as the projection fiber (Fig. 7, upper panel) evaluation (see above).

#### Differential PFC connectivity contribution to (disease) sub-networks

On the projection fiber level vlPFC, dACC and STN appear to act as evolutionary drivers of network contribution changes. With respect to the STN dmPFC, vACC and dACC swap their roles with vlPFC (dominant in the marmoset) indicating an expansion of cognitive motor control (CMCN) on the evolutionary trajectory of PFC development. During species development, STN appears to gain a more important role in delaying the decision making process (Frank et al. 2007; Teichert et al. 2014). Thus phylogenetically, the STN seems to develop into a *bridgehead* of human PFC as an integral part of an expanding PFC and CMCN. With regards to reward and maintenance functions (RMN), VTA shows less connectedness to dACC in humans with a yet relatively stronger connection to the vlPFC. PFC regions which in the marmoset are more closely connected to the VTA as subcortical basis of the *reward maintenance sub-network (RMN)* in humans become more closely associated with the STN *(CMCN)*. As a consequence, RMN seems to have become entirely re-organized. In a way the influence of reward/maintenance (in marmoset) becomes more regulated by cognitive control processes (humans). The connectivity strength of the *affect sub-network (AN)* appears grossly unaltered.

#### Disease specific alterations of connectivity

Disease specific alterations of human connectivity (Fig. 9) allow for inference on connections of the human PFC and thereby might confirm marmoset hardwiring as its less developed blueprint (Fig. 1B). Changes of connectivity which are related to altered function and disease processes therefore underpin the validity of our holistic PFC connectome analysis (cord plots) which serves to compare marmoset (ATTS) and human (DTI FT) connectivity and the evolutionary transient. OCD specific alterations like increased vlPFC to STN connectivity (left) in a way runs counter to the evolutionary routes described above, functionally pointing to the impulsive decision making processes in OCD (Voon et al. 2017) with a disease related enhanced role of the vlPFC (in OCD) related to CMCN. Likewise, reduced dmPFC to premotor connectivity (bilateral) points towards dysfunction in the CMCN and especially the superior frontal gyrus region, previously described in OCD (Menzies et al. 2007; Coenen et al. 2025) in the context of decision making under uncertainty, again reversing evolutionary developmental effects. Such effects potentially point towards a dysfunction in emotional cortical reappraisal. dlPFC with premotor connectivity appears enhanced (although not significantly) in OCD as a hint towards more complex conscious motor control over behaviour with reduced cognitive flexibility. dlPFC is presumed to have inhibitory effects on motor control, stopping complex behaviour and thus showing compensatory effects in OCD, suppressing compulsions and ritualized behavior (Li et al. 2020). In most imaging studies a general PFC hyperactivity is reported in OCD (Ahmari and Rauch 2022).

### Perspectives for Deep Brain Stimulation

Our results show divergent midbrain-PFC connectivity across species that match disease specific alterations of PFC connectivity in patients with severe OCD (Fig. 9) indicating that parts of PFC functionality are compromised in OCD (Ahmari and Rauch 2022). DBS clearly is an effective treatment option for severe OCD that operates within these exact networks (Kohl and Baldermann 2018). Specifically, DBS of the anteromedial STN has shown to be efficacious in alleviating the clinical syndrome of OCD (Mallet et al. 2008, 2019; Chabardes et al. 2020). One might argue that in this respect DBS of the amSTN may reverse a vlPFC to STN relative hyper-connectedness, altering its (functional) connectivity in the direction of an evolutionary trajectory, re-installing STN’s physiological function and thereby dampening impulsive decision making (Voon et al. 2018) and motor program execution (Kibleur et al. 2016). Indeed, previous imaging studies of both DBS to the STN (Voon et al. 2017) and to the IC (Schüller et al. 2023) show that DBS for OCD increases decision impulsivity. Thus, our observed disease-specific alterations of PFC connectivity may functionally correspond to these observations.

## Limitations

We here investigate replications of main streamlines (and signal) of association fibers and descending projections. Single axons have not been analyzed in detail. We compare two different technologies for fiber tract detection (AAVaTT, HCP DTI-FT) in different species each, an approach with certain limitations. Clearly, an interspecies comparison of DTI on the one hand and AAVaTT injections on the other would have been warranted. Especially for DTI, the idiosyncrasies have been mentioned (lack of directionality, lack of transmitter specificity). However, since most projection fiber anatomy is unidirectional (especially the here regarded descending PFC glutamatergic projections to STN, SN and VTA), we do see the feasibility of such a direct comparison (an exception clearly are fibers between PFC and MDT which have known bidirectional routes). Previous work has addressed the comparison of AAVaTT and DTI-FT in the marmoset directly (Skibbe et al. 2023). The authors found “remarkable similarities” between diffusion MRI (dMRI) tractography and anterograde tracer data. Both methods showed tracts passing the internal capsule in segregated streams and projecting strongly to the mediodorsal thalamic nucleus. However, some cortical projections are strong in tracer data but underrepresented in dMRI (false negatives of DTI), whereas some other apparent connections in dMRI are not supported by tracer data (false positives). The work of (Jbabdi et al. 2013) suggests that DWI approaches fail in detection of smaller fibers (like the sub-ICA branch we have described) especially when they penetrate grey matter. We thus cannot entirely solve the sub-ICA dissimilarity between marmoset and human. While we fully appreciate these limitations, we do interpret them as only minimally affecting our large scale and holistic fiber connection analysis. Marmosets share several anatomical and functional characteristics with humans, making them suitable for comparative studies in neuroscience and evolutionary anthropology and the marmoset’s brain organization, including the PFC, provides insights into the evolutionary aspects of primate brain development (Preuss 2019). Especially compared to rodents, the marmoset is considered a valuable model for studying the PFC due to its closer evolutionary relationship to humans. However, while marmosets share some anatomical features with humans, the granular PFC, which is a significant part of the human frontal lobe, is more developed in the latter and other simians than in marmosets. While marmosets can provide useful insights, especially in comparative studies, they will, however, not fully replicate the complexity of the human PFC (Preuss and Wise 2022) which, in turn, makes this species especially useful from an evolutionary research driven research perspective. Disease specific investigations (OCD) in this work should be considered carefully with respect to their interpretation with a rather small - albeit severely affected - patient group (n = 12) at hand.

## Conclusion

The human PFC is specialized for delayed (strategic) decision making processes and facilitates flexible behaviour by integrating exteroceptive sensory and interoceptive stimuli including complex information like emotions, memory, theory of mind and mentalizing. Additionally, the PFC makes the human a “mental time traveler“ relating current information to past, present and anticipated future events (Suddendorf and Corballis 1997). Many of these functions are disturbed in neuropsychiatric diseases like OCD, MDD, Parkinson’s disease and others. Alterations in disease specific sub-network connectivity have been proposed as causative. Previous work has already investigated interspecies PFC connectivity typically utilizing macaque vs human (Croxson et al. 2005; Milham et al. 2018) which are 25 MYA. We here do to look further back in evolution utilizing a new world monkey species 40 MYA from humans (Fig. 1A) with a focus on cortico-cortical and cortico- to subcortical connectivity and psychopathological networks utilizing standard anatomical spaces for group level analyses. With respect to the cortico-subcortical evolution vector, we identified a pronounced connectivity of the subthalamic nucleus with an inferred prominent role of the STN in PFC function regulation presumably with respect to delayed decision making. It indicates an evolutionary transfer of ancient vlPFC function remits towards the STN which might in part be reversed through disease specific alteration of subnetwork connectivity (OCD). Similarly, dmPFC to premotor connectivity is attenuated in OCD (bilaterally) again suggesting maladaptive functional mechanisms which appear to run counter to the evolutionary chosen route of PFC development. Such processes have been discussed to explain vulnerabilities for developing certain diseases (in our case OCD) (Nesse 2023). The mere plausibility of these disease specific results in context with the chosen TT vs DTI approach point toward a validity of our holistic interspecies comparison of PFC connectivity while limitations do apply. Future work should try to corroborate the findings by additional DTI investigations of marmoset connectivity. *Our results allow us to formulate an advanced and phylogeny based hypothesis of psychopathological networks alterations for further verification: Disease pathologies might be interpreted as maladaptations (*Nesse 2023*) toward ancient PFC hard wiring strategies which occurred as older solutions during early evolution. DBS in turn might revert such maladaptations by modulating strategic hub regions like the STN and functionally reactivate an already evolved human PFC hard wiring pattern*. Understanding of the extended connectome of the human PFC is in line with current imaging research and with the STN’s prominent role as a DBS target for neuropsychiatric diseases like Parkinson’s disease and OCD.

## Supplementary Material

Supplementary Files

This is a list of supplementary files associated with this preprint. Click to download.

- supplementalMarmosetnetworks.docx

## Figures and Tables

**Table 1:** Sub-networks in Obsessive-Compulsive Disorder (and major depression disorder): Cortical and subcortical hub regions.

sub-networks*	cortical regions*	Brodman areas+	subcortical regions conjugated with cortical sub-network**
**affect network (AN) (sub-) network**	OFC, vmPFC, vlPFC	8, 9, 10, 11, 13, 14, 12/47 $,**	mdT; AMY; Hipp
	INS	n.N. (anterior/posterior Insula)	
	vACC	25	
**cognitive motor control (sub-) network (CMCN)**	OFC, vmPFC, vlPFC	10, 11, 13, 14, 12/47	AMY, STN, SNr, RN, vlT
	dmPFC, dlPFC	(8), 9, 46 §	
	dACC	32	
	vACC	24, 25	
**default mode (sub-) network (DMN)**	mPFC	8, 9, 10, 24, 25, 32%	-/-
	PCUN	7	
	ANG	40+	
	PCC	31	
**reward/maintenance (sub-) network (RMN)**	PFC (dlPFC, dmPFC, vmPFC, vlPFC, OFC)	8, 9, 10, 11, 12/47, 13, 14, 24–25, 32	NAC, Caudate, VTA

OFC, orbitofrontal cortex; vmPFC, ventromedial prefrontal cortex; vlPFC, ventrolateral prefrontal cortex; INS, insula; vACC, ventral anterior cingulate cortex; dmPFC, dorsomedial prefrontal cortex; dlPFC, dorsolateral prefrontal cortex; dACC, dorsal anterior cingulate cortex; mPFC, medial prefrontal cortex; PCUN, precuneus; ANG, angular region; PCC, posterior cingulate cortex; mdT, mediodorsal thalamus; AMY, amygdala; Hipp, hippocampus; STN, subthalamic nucleus; SNr, substantia nigra; RN, red nucleus; vlT, ventrolateral thalamus; NAC, nucleus accumbens; VTA, ventral tegmental area.

* according to (Li et al. 2018);

+ (Brodmann 1909);

** (Coenen et al. 2020);

§ (Jung et al. 2022);

$ (Lacerda et al. 2003)

% (Petrides et al. 2012)

**Table 2:** Assignment of cortical fields to cortical regions

cortical regions	Human (Huang et al. 2022)	Marmoset (Skibbe et al. 2023)
OFC	10r, 10v, 10pp, 11l, 13l, 47m, 47s, a10p, OFC	A10, 13L, 13M, 11, 10 (6 injections)
dlPFC	10d, p10p, 44, 45, IFJa, IFJp, IFSa, IFSp, p47r, 46, 8Ad, 8Av, 8C, 9-46d, 9a, 9p, a9-46v, i6-8, p9-46v, s6-8	8AD, 8AV, 46V, 9 (29 injections)
vlPFC	47l, a47r	45, 47O (2 injections)
dmPFC	6ma, SCEF, 9m, 8BL, SFL	8B (3 injections)
dACC	8BM, a24, a32pr, d32, p24	32 (3 injections)
vACC	24dd, 24dv, 33pr, a24pr, p24pr, p32pr	24A, 24B, 25 (3 injections)
(pre)motor	24dd, 6mp, 55b, 6a, 6d, 6r, 6v, FEF, PEF	4, 6 DR, 6DR, 6VB, 6M (5 injections)

## Data Availability

Data can be made available upon reasonable request to the corresponding author (vac).

## Abbreviations

AAVaTT: adeno-associated virus anterograde tract tracing
ACC: anterior cingulate cortex
AN: affect (sub-) network
ATTS: anterograde tract tracing streamline tractography
CMCN: cognitive motor control (sub-) network
CSA: connection strength analysis
dACC: dorsal ACC
DBS: Deep Brain Stimulation
dlPFC: dorsolateral PFC
DMN: default mode (sub-) network
dmPFC: dorsomedial PFC
DTI: diffusion tensor magnetic resonance imaging
FT: fiber tractography
HCP: human connectome project
ICa: Internal capsule, anterior limb
MDD: major depressive disorder
mdT: mediodorsal thalamus
MYA: million years apart
NHP: non-human primate
OCD: obsessive-compulsive disorder
OFC: orbitofrontal cortex
PD: (idiopathic) Parkinson’s disease
PFC: prefrontal cortex
RMN: reward maintenance (sub-) network
ToM: theory of mind
TRD: treatment resistant depression
vACC: ventral ACC
vmPFC: ventromedial PFC
vlPFC: ventrolateral PFC
vlT: ventrolateral thalamus
